# A global CORDEX-based dataset delineating urban areas and their surroundings to assess climate change in megacities

**DOI:** 10.1038/s41597-025-06257-1

**Published:** 2025-12-18

**Authors:** Javier Diez-Sierra, Yaiza Quintana, Gaby S. Langendijk, Josipa Milovac, Matthias Demuzere, Rita Nogherotto, Joni-Pekka Pietikäinen, Diana Rechid, Natalia Zazulie, Silvina A. Solman, Jesús Fernández

**Affiliations:** 1 Instituto de Física de Cantabria (IFCA), CSIC-Universidad de Cantabria, Santander, Spain; 2 Climate Adaptation and Disaster Risk Department, Deltares, PO Box 177, 2600 MH, Delft, the Netherlands; 3 B-Kode VOF, Ghent, Belgium; 4 The Institute of Atmospheric Sciences and Climate (CNR-ISAC), Bologna, Italy; 5 The Abdus Salam International Centre for Theoretical Physics (ICTP), Trieste, Italy; 6 Climate Service Center Germany (GERICS), Helmholtz-Zentrum Hereon, Fischertwiete 1, 20095 Hamburg, Germany; 7 Departamento de Ciencias de la Atmósfera y los Océanos, Facultad de Ciencias Exactas y Naturales, Universidad de Buenos Aires, Consejo Nacional de Investigaciones Científicas y Técnicas, Buenos Aires, Argentina; 8 Departamento de Ciencias de la Atmósfera y los Océanos, Facultad de Ciencias Exactas y Naturales, Universidad de Buenos Aires, Centro de Investigaciones del Mar y la Atmósfera, Consejo Nacional de Investigaciones Científicas y Técnicas, Instituto Franco-Argentino de Estudios sobre el Clima y sus Impactos (IRL 3351 IFAECI/CNRS-IRD-UBA), Buenos Aires, Argentina

**Keywords:** Projection and prediction, Climate-change adaptation

## Abstract

We present a global dataset of urban areas and their rural surroundings, developed within the framework of the CORDEX Flagship Pilot Study on Urban Environments and Regional Climate Change. The dataset is derived from model-specific urban fraction variables and additional static inputs. Urban and rural surrounding areas are delineated using Regional Climate Model (RCM) simulations from the global CORDEX-CORE and European EURO-CORDEX initiatives, focusing on a representative set of megacities worldwide. The analysis was conducted at horizontal resolutions of 25 km globally and 12.5 km for Europe. To facilitate future applications, we provide a Python-based workflow that can be extended for the analysis of additional cities and RCMs, including tools for evaluating the urban climate island effect. The dataset and tools, available via Zenodo and GitHub, offer a consistent and reproducible approach for assessing urban climate change in current and upcoming regional climate projections. This constitutes the first global RCM-based database of urban/rural areas, providing a foundation for future high-resolution model data analysis efforts, such as studies using convection-permitting simulations.

## Background & Summary

Understanding how climate conditions vary within urban areas has important implications for stakeholders developing adaptation strategies^1^. The Urban Heat Island (UHI) effect refers to the phenomenon whereby urban areas experience significantly higher temperatures than their surrounding suburban and rural regions^2,3^. This effect can lead to several negative consequences, including increased heat stress and health risks, higher energy consumption, decreased labor productivity and elevated air pollution levels, among others^4–8^. In addition to temperature, dense urban environments can influence other climate variables. For instance, precipitation may increase over and downwind of highly urbanized areas^9–12^; relative humidity is often lower in urban areas compared to rural locations^13–16^; and wind speeds can be significantly altered in cities relative to their surroundings^17–20^.

From a climate perspective, the UHI effect amplifies the frequency, duration, and intensity of heat waves beyond what is typically driven by climate change alone^1^. Consequently, understanding the combined impact of urban climate, urbanisation and climate change on cities worldwide is particularly relevant, especially given that a significant portion of the global population resides in urban areas which is expected to increase in the coming decades^21^. Assessing climate change at regional or global scales requires the use of numerical climate models^22,23^. Two main modeling approaches have emerged for understanding and analyzing the urban climate^24^. First, meso- and microscale urban climate models resolve climate processes at the street-to-city scales (1 m to 1 km). These models simulate short-duration weather events, potentially under climate change conditions, for instance either by adding a specified temperature increase to the model^10,25,26^, or using boundary conditions from Global Climate Models (GCM) or Regional Climate Models (RCM)^10^, or through statistical downscaling methods^27–29^. However, due to limitations in domain size, these models often struggle to fully capture the dynamic interactions between the city and its regional surroundings, as well as the combined effects of climate change and urbanization over climatological timescales^30,31^. Second, RCMs have undergone an increase in grid resolution in recent years, going down to the kilometer scale (1–4 km), which allows them to resolve smaller scale processes and features of the Earth’s surface^32,33^. These so-called convection-permitting RCMs (CPRCM) represent a larger proportion of grid boxes categorized as “urban”, often with higher urban fraction values. They show strong potential for simulating urban climates over long timescales, from decades to even a century^34,35^. The representation of urban areas in RCMs varies in complexity^36^, ranging from simple bulk urban parameterizations^37^, to single-layer urban canopy models^38^, to more advanced multilayer models such as the Building Effect Parameterization/Building Energy Model (BEP/BEM)^39^. In parallel with the development of CPRCMs, several modeling centres have started to develop hectometric-scale weather and climate models for operational use^40^.

Unfortunately, the enormous computational cost inherent to the CPRCMs limits their application to relatively small geographic areas, making it particularly challenging to perform global-scale analyses^41,42^. On the other hand, the coarse spatial resolution (~100 km) of long-term GCM ensemble simulations often prevents these models from capturing urban areas^16,30,43^. Fortunately, RCM projections from initiatives such as the COordinated Regional Downscaling EXperiment – COmmon Regional Experiment (CORDEX-CORE)^44–48^ and EURO-CORDEX^49–52^ provide an opportunity to analyze urban climate change at relatively finer horizontal resolutions: 25 km over all continental CORDEX domains and 12.5 km over the European domain. Despite these advancements, simulations from the CORDEX experiments still face notable limitations: urban schemes are often deactivated^30^, spatial resolution remains insufficient to fully capture urban processes, the representation of urban phenomena is often incomplete or too simplistic^43,53^, and land use changes including urbanisation or greening of cities are not considered to date. Nevertheless, CORDEX projections remain the only globally available source of regional climate change information based on dynamical downscaling at the time, and they have been used as a reference for the regional analyses in the Intergovernmental Panel on Climate (IPCC)’s Sixth Assessment Report^54^. CORDEX-CORE simulations enable the climate research community to evaluate their limitations in representing urban environments across the globe and provide a critical foundation for improving future modeling frameworks^55^. Looking ahead, new CORDEX simulations nested into Coupled Model Intercomparison Project Phase 6 (CMIP6) projections^56^ and particularly the next generation of RCM ensembles at convection-permitting resolutions^32,57–59^, along with advanced downscaling techniques based on deep learning^28^, present a promising framework for future analyses.

In this context, the CORDEX Flagship Pilot Study (FPS) on URBan environments and Regional Climate Change (URB-RCC) was launched in May 2021, with the aim of investigating the impact of cities on regional climates and vice versa^24^. One of the key objectives of the FPS URB-RCC is to assess the capability of existing CORDEX-CORE simulations to represent urban climates. To this end, a selection of representative cities worldwide was made based on various criteria, ensuring a diverse and globally-relevant sample of urban areas^55^. Langendijk *et al*.^55^ show that, although limited, CORDEX-CORE models are capable of reproducing an urban imprint across various megacities. Their findings indicate that the models’ ability to simulate the UHI effect improved significantly when more sophisticated urban schemes were implemented and when the spatial resolution was increased from 25 km to 12.5 km, as in CORDEX-EUR-11.

From the perspective of current and emerging initiatives aimed at providing regional climate change simulations, it is essential to establish a robust and consistent methodology for defining urban areas and their rural surroundings. This methodology must be applicable across different spatial resolutions and adaptable to the specific geographic characteristics of each city, following a land-based approach. Consequently, the methodology relies on a set of input-model static variables to characterize urban and rural areas within the RCM spatial framework, such as urban fraction (i.e., the percentage of the grid cell occupied by urban area), orography, and the land-sea mask, although additional variables could also be incorporated to assess the effect of urbanisation and land use changes. Such an approach is necessary because the representation of urban land cover in numerical climate models often diverges from administrative city boundaries due to the coarse horizontal resolution and the use of varying underlying land cover datasets.

This article presents: (1) a database of urban areas and their rural surroundings for CORDEX-CORE (and CORDEX-EUR-11 for the European domain), covering the cities selected within the framework of the CORDEX FPS URB-RCC, which represents the first global resource for evaluating urban climate change based on two different RCMs; and (2) a Python-based workflow for delineating urban areas and their rural surroundings, which includes additional functionalities for analyzing and visualizing the UHI effect. These tools are specifically designed to be applicable to numerical climate model outputs. The overall objective of this work is to establish a collaborative and consistent framework for assessing urban climate change using reproducible methodologies.

## Methods

### Input data

#### CORDEX simulations

CORDEX (https://cordex.org) represents the first global initiative, established under the auspices of the World Climate Research Programme (WCRP), to coordinate high-resolution regional climate projections within a unified experimental framework^46,60^. CORDEX provides spatially detailed climate change projections from a large ensemble of RCMs applied over large continental areas, at horizontal grid spacing ranging from 12 to 50 km.

In this study, CORDEX-CORE simulations^45^ are used to define urban areas and their rural surroundings areas for selected cities, as part of the first phase of CORDEX FPS URB-RCC (see the Section “Methods”). CORDEX-CORE simulations constitute, to date, the only ensemble of RCMs provided under a common protocol that cover nearly all continental areas of the world (across nine CORDEX domains) at a horizontal resolution of 25 km. The remaining CORDEX-CMIP5-based simulations were performed at coarser resolutions, typically 50 km for most domains, except for Europe, where a higher resolution of 12.5 km is available. The simulations include two RCMs, RegCM and REMO, at a spatial resolution of 25 km (0.22°). To complement this, CORDEX-EUR-11 simulations, with the same RCMs (i.e. REMO and RegCM) but at a higher resolution of 12.5 km (0.11°), are employed for the European cities. The availability of data at both 25 km and 12.5 km resolutions enables a comparative assessment of the added value of increased horizontal resolution in capturing urban climate features. This study focuses exclusively on simulations of the evaluation scenario nested to ERA-Interim reanalysis^61^. Further information on the specific RCM versions used for each domain is provided in Langendijk *et al*.^55^.

The regional climate models RegCM (several variants of RegCM4) and REMO (REMO2015) represent urban areas differently. The REMO version (REMO2015) treats urban surfaces as purely sealed/impervious areas, whereas RegCM employs the CLM Urban (CLMU) model, a single-layer urban canopy model in which the urban fraction is further decomposed into three classes (see Langendijk *et al*.^55^ for a detailed description of the RCMs). For consistency, we aim to align the representation of urban areas across models. In REMO, urban areas are represented as rock surfaces simulating the impervious characteristics of cities, whereas RegCM includes an urban land unit within each grid cell that encompasses both impervious and pervious areas. The different urban densities in each land unit have different impervious area fractions. To enable comparison with REMO, the total impervious surface within each RegCM grid cell is extracted (see Langendijk *et al*.^55^). This study provides both the rural/urban database derived from the original urban area fraction variable (*sfturf*) and from the impervious area fraction (*sftimf*). All analyses presented in this article are based on the *sftimf* variable; however we refer to it generically as the “urban fraction” (UF) to avoid confusion, as this is the commonly used term for the variable.

#### Selected cities

A subset of 41 cities, representing a diverse and heterogeneous sample of urban areas worldwide, was selected following the work done in the CORDEX FPS URB-RCC and forms the basis of the global dataset of urban areas and their rural surroundings developed in this study (see Fig. 5). Given the relatively coarse resolution of CORDEX-CORE (25 km), only large urban areas were included. The selection criteria consider city size, geographic characteristics (e.g., coastal, inland, mountainous, or regions with complex terrain), global balance across CORDEX domains, climate characteristics, and climate impact. A detailed description of the selection criteria and the selected cities is provided in Langendijk *et al*.^55^. Note that for RegCM a 40% cut-off value applied to the urban fraction, as well as some CORDEX domains (i.e. NAM-22 and EAS-22) do not include urban areas. This cut-off implies that cells with less than 40% urban coverage are not classified as urban, effectively excluding most cities. An exception is the EUR-11 domain, where no cut-off value is used.

#### Static (time-invariant) variables

Urban or impervious fraction, orography, and land area fraction are the static (time-invariant) variables used in this work as input data to delineate urban and surrounding areas for cities around the world. Orography and land area fraction are part of the mandatory core set of model output variables defined in the CORDEX-CMIP5 downscaling protocols and are therefore publicly available through the Earth System Grid Federation (ESGF; https://esgf-metagrid.cloud.dkrz.de/search)^62^. In contrast, the urban fraction variable was neither designated as a core model output, nor included in Tier 1 (core or mandatory) or Tier 2 (optional or additional), and is only available upon request from the modeling centers. Fortunately, in the upcoming CORDEX-CMIP6 experiment, the urban fraction (*sfturf*) variable is classified as Tier 2, enhancing the FAIR principles^63^ and enabling future analyses of urban climate with a larger ensemble of models.

In this work, we use urban and impervious area fractions from Langendijk *et al*.^64^, who collected (and post processed) them from the REMO and RegCM CORDEX-CMIP5 modeling centers and made them publicly available on Zenodo (10.5281/zenodo.15700267)^64^. This is the first time that such urban fraction data (and derived impervious data for RegCM) have been made publicly available for CORDEX RCM data at both global and EURO-CORDEX scales. The dataset, provided in NetCDF (Network Common Data Form) format, complies with both the CORDEX archive specifications^65^ and the Climate and Forecast (CF) metadata conventions. This dataset, together with the workflow presented in this study, enables the urban climate research community to investigate urban climate under future climate conditions using CORDEX simulations based on a minimal ensemble of two RCMs within a consistent and comparable framework.

### Algorithm for delineating urban areas and their surroundings

Most studies analyze the UHI effect using satellite-derived land surface temperature (LST) and land use and land cover (LULC) data^66,67^. However, urban representation in numerical climate models often differs from administrative city boundaries due to their typically coarse horizontal resolution and the simplification of LULC categories into a limited set of types interpretable by the models. Additionally, land surface representations vary among RCMs, adding further complexity to their interpretation and intercomparison^68^.

A common approach for delineating urban areas is the City Clustering Algorithm (CCA), developed by Rozenfeld *et al*.^69^, which predicts city growth based on population data. This method has been widely applied in UHI studies because it effectively captures the spatial extent of urban areas. However, since population data are not used as a parameter in climate models, this algorithm can be applied using land-use data instead^70–72^. The CCA utilizes a parameter to define the maximum distance at which grid cells are considered connected and belong to the same urban cluster^73^. Previous studies utilizing climate model outputs typically define a city as the grid cell within the model that is nearest to its center^74,75^.

To define rural surrounding areas, a common method involves generating consecutive layers of cell-width buffers around the urban cluster. The most widely used approach is the Boundary Generation Algorithm (BGA)^73^, which iteratively expands a rural buffer around the city until it reaches an area approximately equal to that of the urban region. Simpler methods often focus on individual cities rather than applying a consistent domain-wide methodology. In such cases, urban areas are identified within a predefined region using LULC-based thresholds, while the remaining grid cells are classified as rural –either explicitly or based on distance-based approaches^35,76–79^.

Although approaches such as the CCA and BGA algorithms are widely applied for delineating urban and rural areas, certain parameters (e.g., the maximum distance at which urban grid cells are considered connected in CCA, or the definition of potential areas for rural expansion in BGA) can significantly affect the results, particularly given the coarse horizontal resolution of RCMs, which often necessitates city-specific adjustments to achieve an accurate representation of urban areas.

#### Algorithm description

The methodology proposed in this study relies on three static variables commonly available in most RCM outputs: urban fraction (sfturf or sftimf), orography (orog), and land area fraction (sftlf). The algorithm can be briefly described as follows. A minimum threshold for the UF determines the grid cells representing the city in the model. Potential rural surrounding areas are then determined based on three main criteria: (1) grid cells must have UF values below a specified threshold; (2) large water bodies (lakes, oceans, and rivers) are excluded via a minimum land area fraction threshold; and (3) grid cells with an elevation difference above a threshold with respect to the urban area are excluded to avoid the effect of altitude on temperature (i.e., adiabatic lapse rate). Grid cells complying with these criteria are selected as candidate rural surroundings areas. The final rural surrounding area is obtained from this candidate grid cells through an iterative morphological dilation process expanding outward from the urban cells. Iterations stop when the number of rural cells reaches a predefined ratio relative to the number of urban cells.

Along with the static variables (UF, orog and sftlf), the algorithm uses several parameters (see Table 1) to determine which grid cells are classified as urban or rural. First, the location of the city of interest (“*lon_city”* and “*lat_city”*) and the study area boundaries of a larger area surrounding the city (“*lon_lim”* and “*lat_lim”*) must be defined in geographic coordinates. Only grid cells within the predefined study area are eligible to be selected as either urban or rural. Then, the UF threshold (“*urban_th”*) determines which grid cells are classified as urban. Cells with UF values greater than this threshold are considered urban cells. Urban areas not connected to the city’s core can be excluded using the “*min_city_size”* parameter. This filters out urban clusters, classifying them as neither urban nor rural, and retains the main urban cluster nearest to the coordinates defined by “*lon_city”* and “*lat_city”*. A threshold for the urban fraction in the surroundings (“*urban_sur_th”)* is used to create a buffer zone around the urban area. Cells with UF values between “*urban_sur_th” and* “*urban_th”* may be influenced by the urban climate and, therefore, are excluded from the rural mask. This parameter is particularly relevant for high-resolution climate models, where relatively high values of “urban_th” can be used and, thus, significant urban fractions might affect the rural surroundings.

**Table 1:** Description of the hyperparameters implemented in the algorithm.

Hyperparameter	Description
*“lon_city”* and “*lat_city”*	Longitude and latitude of the city center.
*“lon_lim”* and “*lat_lim”*	Geographic boundaries of the study area (incl. city surroundings) relative to the city center (“*lon_city”* and “*lat_city”*). Grid cells outside these limits (lon_city ± lon_lim and lat_city ± lat_lim) are excluded from the analysis.
*“urban_th”*	Urban fraction threshold (%). Grid cells with urban fraction values above this threshold are classified as urban cells.
*“urban_sur_th”*	Urban surrounding threshold (%). Grid cells with urban fraction values below this threshold are candidates for rural surroundings. Defaults to “urban_th”.
*“orog_diff”*	Maximum elevation difference (in meter) relative to the range (max-min) urban cell elevations (urban min elev. - orog_diff < rural elev. <urban max elev + orog_diff). Pixels exceeding this difference are excluded.
*“sftlf_th”*	Minimum land area fraction (%) required to include a grid cell in the analysis.
*“min_city_size”*	Minimum size (in number of edge-connected cells) for urban clusters to be retained. Urban clusters are excluded, except for the main cluster nearest to “*lon_city*” and “*lat_city*”, which is always retained.
*“ratio_r2u”*	Ratio of rural to urban grid cells. The iterative dilation process stops once this ratio is achieved.

To define potentially rural cells, the algorithm uses orography and land area fraction variables to apply additional filters. The parameter “*orog_diff”* is used to exclude surrounding mountainous areas where elevation difference relative to the minimum or maximum urban cells exceeds a user-defined threshold (in metres). Similarly, large water bodies, such as lakes, oceans and large rivers, are excluded using a user-defined threshold on the land area fraction (“*sftlf_th”).* Note that this parameter also affects the delineation of urban areas. Once candidate rural cells are identified, an iterative morphological dilation process is applied to grow the surrounding area outward from the urban core. The ratio of rural to urban cells is controlled by the “*ratio_r2u”* parameter.

The morphological dilation function is implemented using the scikit-image Python package^80^. This function assigns to a pixel the maximum value found over all pixel values within its surrounding local neighborhood. The neighborhood is defined by a footprint, which is a binary mask (a small matrix of 0 s and 1 s) that specifies the shape and size of the neighborhood by indicating which neighboring pixels are included in the operation. Pixels corresponding to 1 s in the footprint are considered part of the neighborhood, while those with 0 s are excluded. Two types of footprints are implemented: a cross-shaped footprint that considers 4-connected neighbors (cells sharing the edge), and a square-shaped footprint that includes both edge- and corner-connected neighbors (8-connected). In each iteration, the cross-shaped footprint is applied first. If no new rural cells are added, the square footprint is then applied. This dual-step approach is necessary because, in some cities, the cross-shaped footprint fails to expand the masks when using coarse-resolution data. In every iteration, grid cells excluded due to elevation, water bodies, or being classified as urban are ignored. The process terminates once the number of rural cells reaches the desired rural-to-urban ratio specified by the “*ratio_r2u”* parameter.

#### Hyperparameters selection criteria

The algorithm presented here includes several hyperparameters that must be adjusted on a case-by-case basis to ensure optimal performance. Most of these hyperparameters, such as “*urban_th”*, “*urban_sur_th”*, “*min_city_size*” and “*ratio_r2u”*, are designed to accommodate different horizontal resolutions, ranging from cases where a city is represented by only a few grid cells typically for coarse spatial resolutions to others where dozens of grid cells represent the city, for instance at fine spatial resolutions. This makes the algorithm suitable for analysing RCM data across spatial scales, and it has been validated for resolutions ranging from 50 - 2 km. Other hyperparameters, such as “*orog_diff” and “sftlf_th”*, depend on the specific geographic characteristics of each city. The city-specific hyperparameters used to generate the urban/rural mask database for CORDEX-CORE and CORDEX-EUR-11 are provided in Table 2.

**Table 2:** Hyperparameters used to generate the global dataset of urban areas and their rural surroundings. Some hyperparameters are common across cities and spatial resolutions, while others are defined ad hoc for each city and are included in the accompanying YAML file available in the GitHub repository referenced in the “Code Availability” section.

Hyperparameter	CORDEX-CORE	CORDEX-EUR-11
*lon_city* and *lat_city*	See YAML file	
*lon_lim* and *lat_lim*	Typically, “lon_lim” = 1 and “lat_lim” = 1, but some cities require higher limits	
*urban_th*	10%	40%
*urban_sur_th*	None	10%
*orog_diff*	Typically, “orog_diff” = 100 m, but some cities require higher limits Typically, “*sftlf_th*” = 70%, but some cities require lower values to include any urban cell (see YAML file)	
*sftlf_th*		
*min_city_size*	See YAML file	
*ratio_r2u*	2	

The representation of urban environments is highly sensitive to both the horizontal resolution of the data and the urban fraction threshold applied. Commonly, studies use an urban fraction threshold (“*urban_th”*) between 10% and 30%^35,81^. A sensitivity analysis of “*urban_th”* was conducted on the sample of cities to determine an appropriate value for the CORDEX-CORE models at a 25 km resolution^55^. For “*urban_th”* >10%, some cities either disappear or are represented by only a single urban grid cell (e.g., Mexico City). Consequently, due to the relatively coarse resolution of the CORDEX-CORE dataset, a UF threshold of 10% was selected for identifying urban grid cells. This choice is consistent with thresholds used in other regional climate modeling studies at similar horizontal resolutions, such as Daniel *et al*.^81^. For CORDEX-EUR-11, which has a higher horizontal resolution (four times as many grid cells as CORDEX-CORE), a higher UF threshold of 40% was applied. This finer resolution also allows the use of the “*urban_sur_th”* parameter to exclude rural cells with intermediate UF values that may still be influenced by urban environments. For CORDEX-EUR-11 cities, “*urban_sur_th”* was set to 10%, thereby excluding suburban areas and smaller settlements around cities with UF values between 10% and 40% from the rural surroundings. Oceans, larger lakes, and major rivers were excluded by applying a land area fraction threshold (“*sftlf_th”*) of 70%. To account for temperature lapse rate effects, surrounding grid cells with an elevation difference of more than 100 meters from the maximum and minimum elevation of urban cells were also excluded (*orog_diff* = *100*). These two parameters (“*sftlf_th”* and “*orog_diff”)* were adjusted in certain cases based on the geographic characteristics of individual cities, though we aimed to keep them as consistent as possible to ensure comparability. Finally, the ratio of rural to urban cells (“*ratio_r2u”*) was set to 2 for both CORDEX-CORE and CORDEX-EUR-11.

The selected hyperparameters for each city are specified in the GitHub repository referenced in the “Code Availability” section. These hyperparameters were used to generate the dataset presented in this study.

## Data Records

### Dataset of urban areas and their surrounding reference rural regions

A dataset of urban areas and their reference rural surroundings has been generated, using the input data (10.5281/zenodo.15700267)^64^ and the algorithm outlined in the Section “Methods”, and published on Zenodo (10.5281/zenodo.17257489)^82^, for the RCMs and cities listed in Langendijk *et al*.^55^. The dataset consists of a series of NetCDF files –for each combination of RCM (REMO or RegCM), input data (*sftimf* or *sfturf*) and city– containing grid-point values of 0, 1, or NaN, representing rural, urban, or unclassified cells, respectively. For the European cities, two separate files were generated for CORDEX-CORE and CORDEX-EUR-11 at a 25 and 12.5 km of horizontal resolution, respectively. The native map projections of each RCM are preserved, and the NetCDF files also include the hyperparameters used in the algorithm (see the Section “Methods”).

**Fig. 1:**
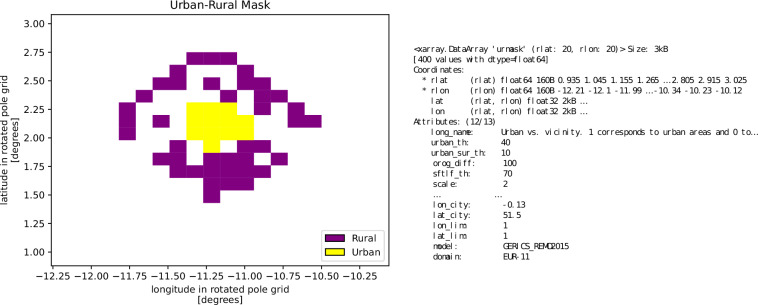
Urban/rural areas for the city of London using the REMO model from CORDEX-EUR-11. Shaded colors represent the values of the variable ‘urmask’, where 1 (yellow cells) indicates grid cells classified as urban, 0 (purple cells) corresponds to rural cells, and NaN denotes areas not classified as either urban or rural. On the right, the dimensions, coordinates, and attributes of the “urmask” variable are displayed.

Filenames and NetCDF metadata are formatted to follow the CORDEX archive specifications^65^ and the CF metadata conventions. Each filename includes the following fields, separated by underscores: “urmask” (Urban/RuralMASK) variable including input data (*sftimf* or *sfturf* depending on the input data used), CORDEX domain including city, driving GCM, experiment, ensemble member, RCM institution, RCM model name, and frequency. For example, the filename for REMO, CORDEX-EUR-11, and London is: urmask-sftimf_EUR-11-London_ECMWF-ERAINT_evaluation_r1i1p1_GERICS_REMO2015_fx.nc. Figure 1 shows a snapshot of the contents of this example, including the hyperparameters used during its generation.

## Technical Validation

An in-depth technical validation was carried out for the data records of each city and RCM combination. The analyses include: (1) urban and rural mask representations; (2) an assessment of the UHI intensity; (3) summary information about the number of grid points classified as urban and rural; and (4) a sensitivity analysis evaluating the impact of interpolation and bias adjustment of the raw data (e.g. temperature) on the resulting UHI intensity estimates.

The GHS-UCDB dataset^83^ offers a globally consistent and harmonized representation of urban centers, making it a suitable reference for validating the urban extent represented in the RCMs. It defines “Urban Centres” as polygons based on population and build-up area thresholds, using data from the Global Human Settlement Layer (GHSL) combined with other open datasets. These centers are mapped on a uniform 1 × 1 km global grid and include various thematic attributes across multiple time periods. While this study uses the GHS-UCDB 2019 version, we acknowledge that a more recent release of GHS-UCDB is available^84^ which may be used for validation in future versions of the dataset.

### Dataset Representation of urban and reference surrounding areas

**Fig. 2:**
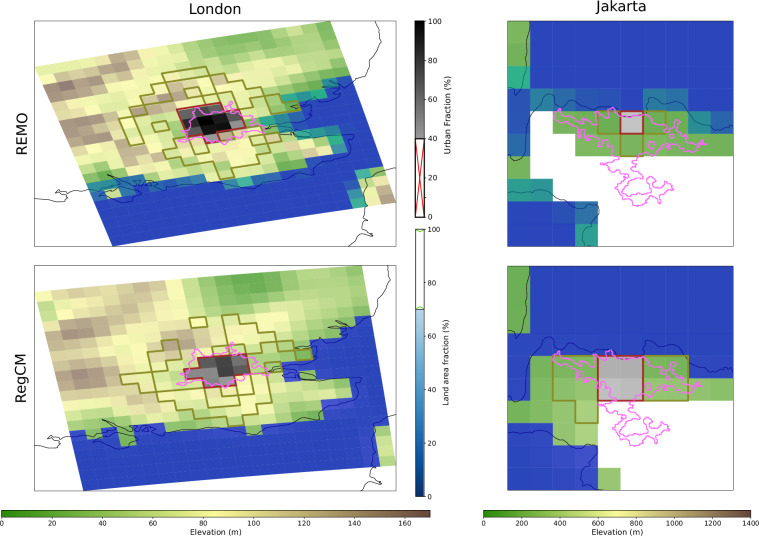
Urban areas (brown polygons) and their rural surroundings (green polygons) for REMO and RegCM models for the cities of London (CORDEX-EUR-11) and Jakarta (CORDEX-CORE). The figure shows the static variables used by the algorithm to delineate urban/rural areas. Orography is represented using a green-to-brown color scale, water coverage is indicated by blue intensity, and urban fraction is shown in grayscale. White-colored cells correspond either to areas with large elevation differences relative to urban areas or to areas beyond the boundaries (“*lon_lim”*, “*lat_lim”*). Urban fraction is displayed only for urban areas (UF >10% for Jakarta and UF >40% for London). The pink polygon indicates the city extent as defined by the GHS-UCDB dataset.

Figure 2 presents examples of the dataset of urban areas and their rural surroundings for London (United Kingdom) and Jakarta (Indonesia), derived from the REMO and RegCM simulations. The hyperparameters used to generate these masks are specified in the corresponding YAML configuration file (see Table 2).

The example illustrates that the representation of urban areas differs substantially between the two models included in the dataset (REMO and RegCM) due to their distinct approaches to representing urban areas (see the Section “Methods”). For London, the extent of the urban area aligns well with that of the GHS-UCDB polygon. However, due to the coarser resolution of CORDEX-CORE, Jakarta is represented by a very limited number of grid cells—only one in the case of REMO—which leads to a significant mismatch with the urban area representation from the GHS-UCDB dataset.

Moreover, the higher resolution of the London example allows the application of the “*urban_sur_th”* parameter, which defines a transitional buffer zone around urban grid cells (see the Section “Methods”). This parameter helps exclude rural cells that are still influenced by the urban climate. A practical demonstration of how this parameter affects UHI intensity is provided in a Jupyter Notebook included in the GitHub repository referenced in the “Code Availability” section.

### Assessment of UHI Intensity

**Fig. 3:**
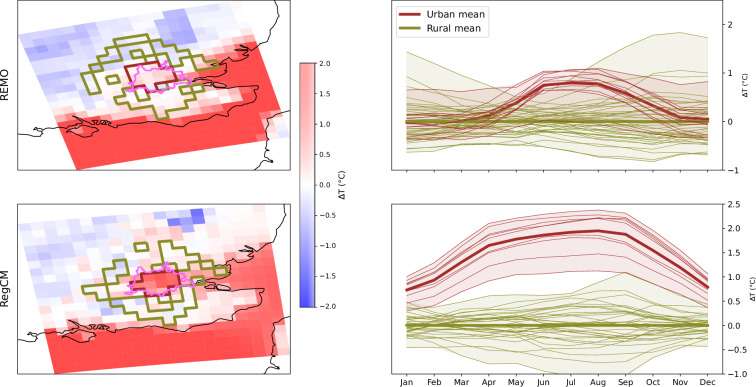
Minimum temperature anomaly maps (relative to the mean rural minimum temperature) for London, based on RegCM and REMO model simulations over the CORDEX-EUR-11 domain. The first column presents spatial maps, where solid brown and green lines represent urban and rural areas, respectively. The pink polygon indicates the city extent as defined by the GHS-UCDB dataset. The second column shows the monthly annual cycle for urban (brown) and rural (green) areas. Each line corresponds to an individual urban or rural grid cell, while thicker lines represent the median across all urban or rural cells. The evaluation period is 1979–2014.

**Fig. 4:**
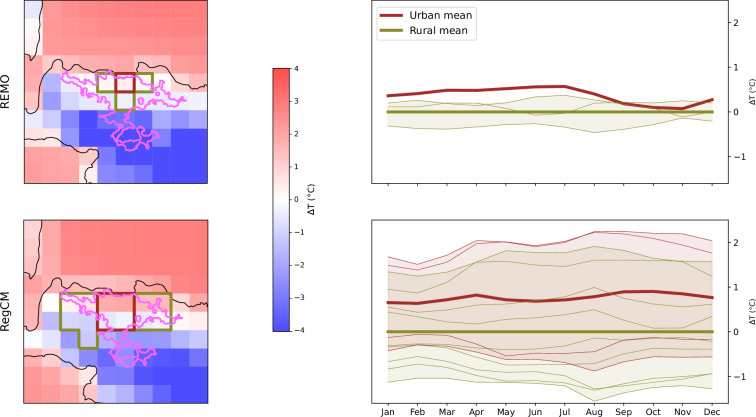
Same as Fig. 3 but for Jakarta using the CORDEX-CORE models.

The dataset allows for assessing the effects of urbanization on climate compared with the reference rural surrounding areas. Figures 3, 4 show the UHI analysis for London and Jakarta (presented as spatial map climatologies and annual cycles) represented as anomalies relative to the mean rural minimum temperature. As shown in Figs. 3, 4 (left panels), there are significant differences in the delineation of urban/rural areas between RCMs at the CORDEX-EUR-11 domain resolution. For CORDEX-EUR-11 simulations (Fig. 3), REMO tends to underestimate UHI intensity compared to RegCM, likely due to the simplified bulk-based urban scheme and limited ability to retain heat during the day and release it at night. These discrepancies become even more pronounced in the CORDEX-CORE domain for Jakarta (Fig. 4), where the coarser horizontal resolution further reduces the UHI intensity. In this case, the distinction between urban and rural grid cells becomes less meaningful, as some exhibit similar temperature anomalies. Note that the UHI in inland cities is typically more intense than in coastal ones; nevertheless, we chose to showcase coastal cities to highlight the challenges associated with the algorithm to accurately delineate urban and surrounding rural areas in coastal environments.

The UHI results from the RegCM model align with previous observational studies. Zhou *et al*.^85^ reported UHI intensities of 0.5 °C to 3 °C for London, while Siswanto *et al*.^86^ found UHI intensities between 1 °C and 2.5 °C for Jakarta. It should be noted that the observational approach to characterizing UHI intensity is highly influenced by intra-urban variability and, therefore, may not be directly comparable to our RCM-based approach^87^. The figures, directly generated using the tools provided in the GitHub repository associated with this study, are also published on Zenodo^88^ for all combinations of cities and RCMs.

### Summary of urban/rural number of grid cells

**Fig. 5:**
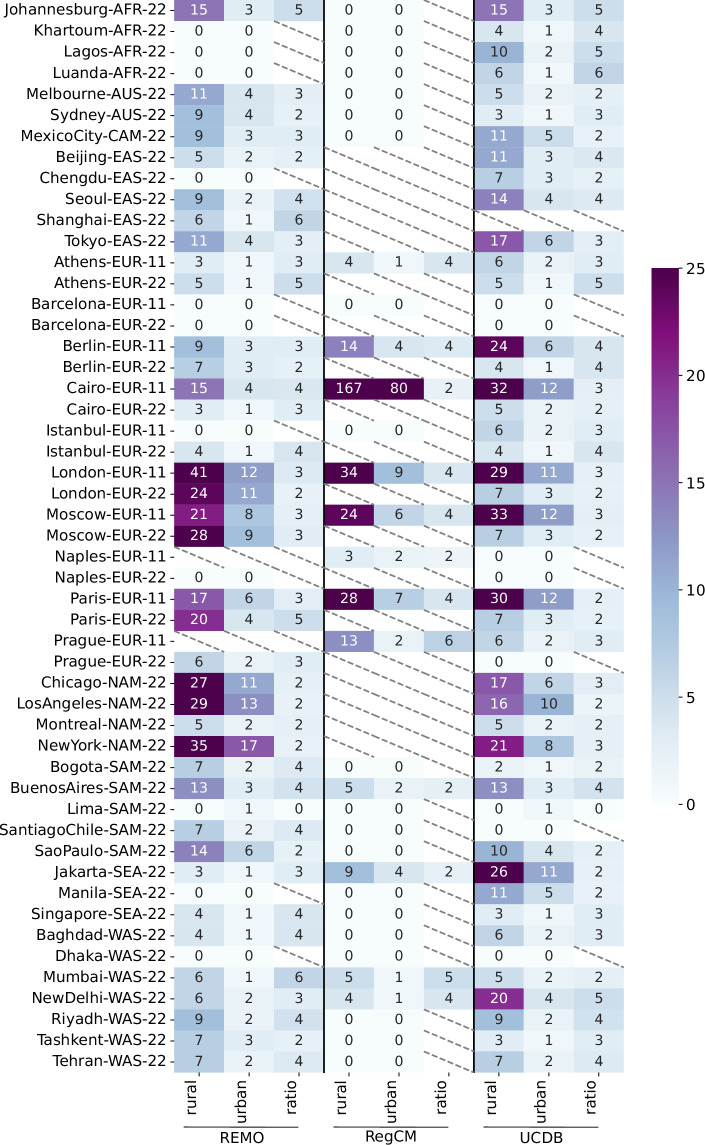
Heatmap showing the number of urban and rural grid cells, as well as their actual ratio of rural to urban grid cells (the target is 2), derived from the REMO and RegCM models, as well as from GHS-UCDB-based dataset. The analysis includes both the CORDEX-CORE (0.22°) and CORDEX-EUR-11 (0.11°) domains. City names are accompanied by their corresponding domain and resolution for reference. A value of zero for urban cells indicates that no grid cell exceeded the urban fraction threshold defined for that model, and therefore, no urban area was detected at this resolution. In contrast, missing values (i.e., empty cells) indicate that urban fraction data are not available for that specific model-domain combination.

Figure 5 shows the number of urban and rural grid cells as well as their ratio for the data records of each city and RCM combination compared with the GHS-UCDB polygons. For the latter, as a proxy, grid cells whose centers fall within the polygon are assumed to have an UF of 100%, ensuring independence from the applied urban threshold. The dilation algorithm is then applied to delineate rural areas following the same approach. The rural-to-urban ratio parameter (“*ratio_r2u*”) is set to 2; however this threshold may be considerably exceeded, as the algorithm completes an entire dilation cycle in each iteration. In contrast, forcing the process to stop precisely when the target ratio is reached would introduce an undesirable dependency on the order in which the outer is traversed. This could lead to directional bias in the expansion of the rural area, undermining the spatial neutrañlity of the method.

Since RegCM does not provide urban fraction information for the EUR-22, EAS-22, and NAM-22 domains; these combinations are therefore excluded from the analysis shown in Fig. 5. Smaller cities such as Khartoum, Lagos, and Manila do not reach UF values above 10%, and thus no urban areas are detected in these cases. Additionally, there are instances where cities do exceed the 10% UF threshold, but their urban areas primarily overlap with water bodies and are therefore excluded (e.g. Barcelona in the EUR-22 domain). Furthermore, the cut-off threshold applied in RegCM for CORDEX-CORE, which retains only urban fractions above 40% in all domains except EUR, results in only four cities being included (Buenos Aires, Jakarta, Mumbai and New Delhi). In general, RegCM underestimates the number of urban grid cells for CORDEX-CORE when compared to the GHS-UCDB dataset, while REMO results are closer to the reference dataset. Conversely, for the EUR-11 domain, REMO tends to underestimate the number of urban cells while RegCM more closely aligns with the GHS-UCDB values. An in-depth assessment of urban land cover for both RCMs is described by Langendijk *et al*.^55^.

### Assessing the impact of interpolation and bias adjustment

The dataset of urban/rural masks is generated deliberately in the native projection of each RCM, avoiding interpolation to prevent the introduction of external artifacts. RCMs may use different map projections depending on their domain (e.g., North America, Africa, Europe) and the climate model itself. Therefore, interpolating raw RCM data onto a regular, common grid is a widespread practice, as it significantly facilitates data analysis. Several methods can be applied for regridding, with two of the most commonly used being conservative remapping and Nearest-Neighbour (NN). The conservative remapping method is typically used to preserve mass and conservation laws, whereas the NN method is a simpler approach that retains the magnitude of the raw data by selecting values from the closest grid point to the target location.

**Fig. 6:**
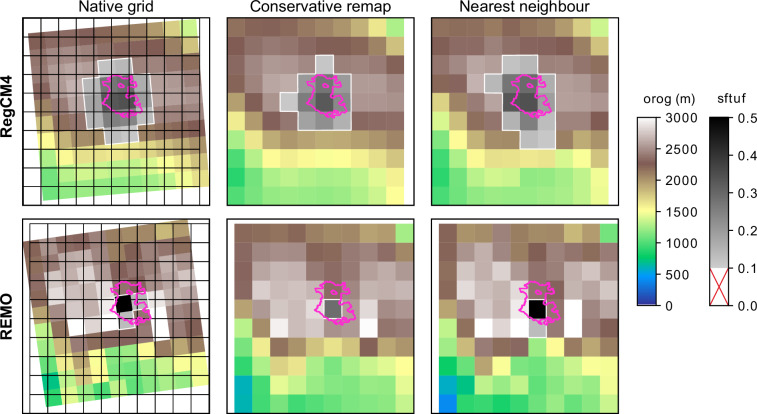
Example of the interpolation effect on urban fraction for Mexico City, using the RegCM and REMO models. The pink polygon corresponds to the GHS-UCDB dataset. The target grid in the last two columns is shown as a black line grid overlaid on the native grid in the first column.

For cities represented by a small number of grid cells (as is the case for most cities in CORDEX-CORE), the choice of interpolation method becomes particularly important. As shown in Fig. 6, the NN method preserves the original magnitude of the variable and, consequently, the UHI signal. However, it has the drawback of potentially shifting the location of the data and, in some cases, altering information from the original dataset. Since the number of target grid cells differs from the number of native ones, the NN mapping drops or duplicates values, regardless of their relevance (e.g. the largest urban fraction could be dropped or duplicated). In contrast, the conservative remapping method smooths the results. To avoid the given limitations, the database and results presented in this work were calculated using the native projection of each model.

When using the dataset for UHI assessment, it is recommended to verify whether the regional model outputs have been bias-adjusted. Bias adjustment of RCMs outputs using a reference observational dataset is a common approach applied to reduce systematic biases in climate model outputs, and their use is recommended for regional applications^89^. However, this approach is only advisable when a high-quality observational reference dataset is available. In the context of urban analyses, the observational reference needs to properly represent urban areas, with a fair amount of observing stations within the city and in the surrounding areas. Otherwise, the UHI signal produced by the RCM might be lost.

**Fig. 7:**
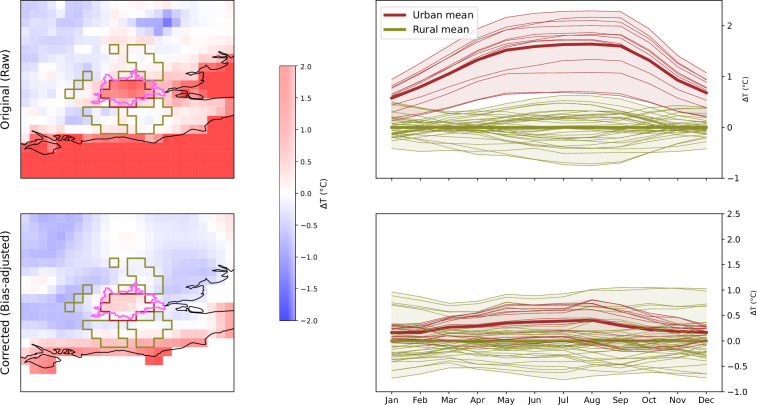
Minimum temperature anomaly maps (relative to the mean rural minimum temperature) for London, based on RegCM model simulations over the CORDEX-EUR-11 domain (see Fig. 3 for more details). The top panels show results obtained from the original (raw) model output, while the bottom panels present bias-adjusted results using E-OBS as reference.

Figure 7 illustrates an example of the minimum temperature UHI climatology for London before and after bias adjusting CORDEX-EUR-11 (RegCM) by means of the widely-used E-OBS dataset^90^ as reference. Both CORDEX-EUR-11 and E-OBS have similar horizontal resolutions: 0.11° and 0.1°, respectively. The results clearly show that the UHI intensity signal in the model almost disappears after bias adjustment while the UHI intensity calculated directly from is closer to the UHI intensities reported in other studies (between 0.5 °C to 3 °C)^85^. These results highlight the crucial role of the reference dataset in such applications.

## Data Availability

The dataset of urban and surrounding reference rural regions is available on Zenodo (10.5281/zenodo.17257489)^82^ under the Creative Commons Attribution 4.0 International.

## Code availability

The Python code, including Jupyter Notebooks, to generate the urban/rural dataset and the additional functionalities to calculate and visualize the urban heat island, are available on GitHub (https://github.com/FPS-URB-RCC/urclimask) and Zenodo (10.5281/zenodo.17257445)^88^. The urban/rural dataset is available on Zenodo (10.5281/zenodo.17257489)^82^ under the Creative Commons Attribution 4.0 International. The version Version v1.0.1 has been used to generate the dataset (10.5281/zenodo.15707502). The selected hyperparameters for each city are specified in the GitHub repository: https://github.com/FPS-URB-RCC/urclimask/tree/main/code/CORDEX-CMIP5/selected_cities.yaml. A Jupyter Notebook indicating how hyperparameters configuration affects UHI intensity is included on GitHub: https://github.com/FPS-URB-RCC/urclimask/blob/main/notebooks/paris_across_CORDEX_resolutions.ipynb.
